# Development and Utility of an Imaging System for Internal Dosimetry of Astatine-211 in Mice

**DOI:** 10.3390/bioengineering11010025

**Published:** 2023-12-26

**Authors:** Atsushi Yagishita, Miho Katsuragawa, Shin’ichiro Takeda, Yoshifumi Shirakami, Kazuhiro Ooe, Atsushi Toyoshima, Tadayuki Takahashi, Tadashi Watabe

**Affiliations:** 1 Kavli Institute for the Physics and Mathematics of the Universe (Kavli IPMU, WPI), The University of Tokyo, 5-1-5, Kashiwanoha, Kashiwa 277-8583, Japan; miho.katsuragawa@ipmu.jp (M.K.); shinichiro.takeda@ipmu.jp (S.T.); tadayuki-takahashi@g.ecc.u-tokyo.ac.jp (T.T.); 2 Institute for Radiation Sciences, Osaka University, 1-1, Yamadaoka, Suita, Osaka 565-0871, Japan; yoshifumi_shirakami@irs.osaka-u.ac.jp (Y.S.); ooe@rirc.osaka-u.ac.jp (K.O.); toyo@irs.osaka-u.ac.jp (A.T.); watabe.tadashi.med@osaka-u.ac.jp (T.W.)

**Keywords:** astatine-211, radiotheranostics, dosimetry, in vivo imaging, quantitative imaging

## Abstract

In targeted radionuclide therapy, determining the absorbed dose of the ligand distributed to the whole body is vital due to its direct influence on therapeutic and adverse effects. However, many targeted alpha therapy drugs present challenges for in vivo quantitative imaging. To address this issue, we developed a planar imaging system equipped with a cadmium telluride semiconductor detector that offers high energy resolution. This system also comprised a 3D-printed tungsten collimator optimized for high sensitivity to astatine-211, an alpha-emitting radionuclide, and adequate spatial resolution for mouse imaging. The imager revealed a spectrum with a distinct peak for X-rays from astatine-211 owing to the high energy resolution, clearly distinguishing these X-rays from the fluorescent X-rays of tungsten. High collimator efficiency (4.5 × 10^−4^) was achieved, with the maintenance of the spatial resolution required for discerning mouse tissues. Using this system, the activity of astatine-211 in thyroid cancer tumors with and without the expression of the sodium iodide symporter (K1-NIS/K1, respectively) was evaluated through in vivo imaging. The K1-NIS tumors had significantly higher astatine-211 activity (sign test, *p* = 0.031, n = 6) and significantly decreased post-treatment tumor volume (Student’s *t*-test, *p* = 0.005, n = 6). The concurrent examination of intratumor drug distribution and treatment outcome could be performed with the same mice.

## 1. Introduction

Radiotheranostics has garnered remarkable attention in nuclear medicine in recent years [1,2,3]. Agents labeled with alpha-ray-emitting radionuclides ( α -emitters) have gained prominence [4,5]. As the radiation dose to tissues correlates with therapeutic and side effects, estimating its distribution and absorbed dose before administration is a standard practice. As the radiation sources in radiotheranostics are radiopharmaceuticals, the radiation dose of a given tissue is primarily influenced by their pharmacokinetics and decay. Importantly, alpha particles are markedly more cytotoxic than X-rays and β -rays [6]; thus, a precise understanding of radiopharmaceutical pharmacokinetics and biodistribution is required for drug development and clinical application. For pharmacokinetic evaluation, the radiation dose of a radiopharmaceutical in animal tissues is often determined by sacrificing several animals at various time points, collecting tissue samples, and plotting time–activity curves for target tissues. The disadvantages of this method include the requirement to sacrifice numerous mice and the inability to monitor them postsacrifice. Moreover, this method is not feasible for human studies due to tissue sample constraints. Quantitative imaging addresses these limitations to an extent.

X/ γ -ray camera imaging enables simultaneous analysis of the distribution of radiopharmaceuticals and their quantification in vivo. This method uses X/ γ -rays emitted by radionuclides for imaging and appears useful as many, if not all, therapeutic radionuclides emit X/ γ -rays available for quantitative imaging. In β -emitter-labeled therapeutic agents, where quantitative imaging is possible to some extent, clinical reports have revealed a correlation between absorbed dose and therapeutic efficacy in certain cancers [7,8,9,10,11]. However, quantitative imaging of α -emitter-labeled therapeutics is challenging. The main reason for this challenge is the low dose for imaging. Due to the higher linear energy of α -rays, the dosage of α -emitter-labeled radiopharmaceuticals is markedly lower than that of diagnostic radiopharmaceuticals. The low intensity of X/ γ -rays suitable for X/ γ -ray camera imaging is also another reason. For instance, the γ -ray intensity of ^99*m*^Tc is suitable for imaging (i.e., the intensity of 141 keV γ -ray) is 89% [12]. In contrast, ^211^At, a promising α -emitter, emits X-rays suitable for imaging (approximately 80 keV of $K_{\alpha }$ lines and approximately 90 keV of $K_{\beta }$ lines); however, their combined intensity is only approximately 40% [13]. The current X/ γ -ray camera and single-photon emission computed tomography (SPECT) are not designed to address these issues, highlighting the need to enhance both sensitivity and the signal-to-noise ratio (SN-ratio) for accurate quantification. In addition, small animal imaging requires a higher spatial resolution than humans due to their smaller size. Higher spatial resolution reduces sensitivity. Therefore, innovations in small animal imaging is essential to maintain adequate sensitivity. In summary, an optimal imager requires a high SN-ratio, suitable spatial resolution for the target, and elevated sensitivity. Augmentation of the SN-ratio requires the utilization of a detector with a higher energy resolution. Furthermore, to enhance sensitivity without an excessive reduction in spatial resolution, it is imperative to establish the minimal spatial resolution requisite and to optimize the thickness and length of the collimator septa, thereby maximizing photon yield.

To address these challenges, we have been developing a planar imager for small animals that have high sensitivity and high SN-ratio. This imager is equipped with a cadmium telluride (CdTe) detector with high energy resolution [14] and a tungsten 3D-printed precision collimator optimized for a specific radionuclide type. ^211^At, which is a promising therapeutic radionuclide that emits α -rays [15], was selected as the target radionuclide for optimizing the collimator. Thus, a collimator tailored for X-rays from ^211^At (the $K_{\alpha }$ and $K_{\beta }$ emission lines of ^211^Po) [16] was fabricated. However, the imaging of ^211^At using cancer cells and animal models has not been validated effectively. This method requires verification in terms of quantification and the practicality of imaging times in biological experiments. Moreover, the utility of imaging At also requires validation, similar to how the imaging of beta-emitting radionuclides has been clinically demonstrated to be effective.

Astatine is a halogen. Moreover, the astatine ion is known to exhibit pharmacokinetics similar to those of the iodide ion (which is also a halogen) [17]. ^131^I-NaI is known to be effective in the treatment of thyroid cancer [18,19,20]. ^211^At-NaAt has shown similar efficacy in this regard, as demonstrated in our previous studies [21,22]. Therefore, an experimental system using ^211^At-NaAt for thyroid cancer is suitable for examining the validity and utility of quantitative imaging.

In this study, we aimed to present the performance of our planar imager, equipped with a further optimized collimator to increase sensitivity. Thereafter, experiments with thyroid-cancer-bearing mice treated with ^211^At-NaAt were performed to evaluate biodistribution using in vivo imaging and highlight the utility and significance of in vivo imaging for therapeutic radiopharmaceuticals.

## 2. Results

### 2.1. Specification of the Imager

**Figure 1:**
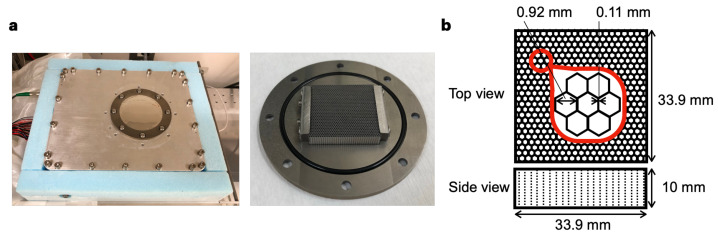
Overview of the imager. (**a**) Exterior appearance of the imager (**left**) and appearance of the collimator (**right**). (**b**) Layout of the parallel-hole collimator.

Figure 1a shows the bench-top-sized planar imager (L 25 × W 20 × H 15 cm). This imager is equipped with a 3D-printed tungsten collimator, which has a thickness of 10 mm, a septa thickness of 0.11 mm, and a hole diameter of 0.92 mm (Figure 1b). The efficiency of the collimator was improved by reducing its thickness from 20 mm reported in our previous study to 10 mm, which resulted in an increase in collimator efficiency from 1.4 × 10^−4^ [16] to 4.5 × 10^−4^. The hole diameter of the collimator was designed to maximize photon collection while maintaining adequate spatial resolution for identifying small organs, such as the thyroid gland.

**Figure 2:**
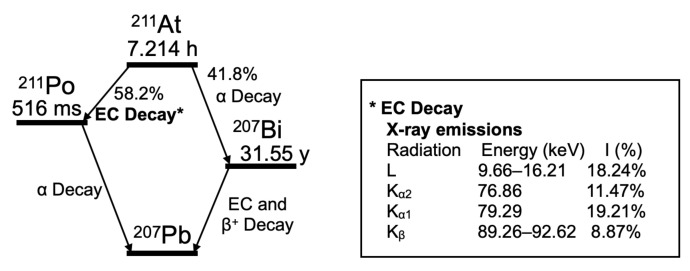
Decay scheme of ^211^At. The $K_{\alpha }$ and $K_{\beta }$ lines of the X-rays derived from electron capture (EC) decay are used for imaging. I (%); intensity per decay (%).

Figure 2 illustrates the decay scheme of ^211^At, based on the decay data standards from the Lund/LBNL Nuclear Data Search, Version 2.0 [13]. The septa thickness was designed to be thick enough to shield the ^211^At X-rays (the $K_{\alpha }$ and $K_{\beta }$ lines of ^211^Po, approximately 80–90 keV). ^201^Tl emits X-rays at approximately 70 keV. It is used clinically as a diagnostic nuclide. Therefore, X-rays of 80–90 keV (which is close to the energy of X-rays of ^201^Tl) are suitable for animal imaging.

### 2.2. Performance of the Imager with Respect to ^*211*^At X-rays

**Figure 3:**
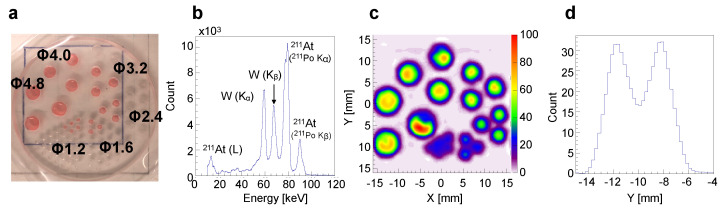
Evaluation of the spatial resolution and spectrum using ^211^At solution. (**a**) Derenzo phantom loaded with ^211^At solution (red). Φ indicates the hole diameter (mm). (**b**) Spectrum obtained from phantom. (**c**) Image generated by using the ^211^At X-rays (the $K_{\alpha }$ emission line of ^211^Po, 75–85 keV). (**d**) Cross-sectional view (count vs. position) through the center of the 1.6 mm-diameter holes in (**c**).

The spatial resolution at approximately 80 keV was evaluated using a Derenzo phantom (Figure 3a) filled with a red-colored solution containing ^211^At anion in each hole. The acquired spectral data (Figure 3b, count vs. energy) revealed distinct spectral peaks from the ^211^At X-rays (the $K_{\alpha }$ and $K_{\beta }$ lines of ^211^Po), which are clearly distinguishable from the fluorescent X-rays of tungsten (W( $K_{\alpha }$ ) and W( $K_{\beta }$ ) in Figure 3b). The phantom image using 75–85 keV (the $K_{\alpha }$ line) photons (Figure 3c) shows that each hole with a diameter of 1.6 mm or larger can be discernible separately. This observation aligns with the cross-sectional view through the center of the 1.6 mm-diameter hole (Figure 3d, count vs. position), which clearly displays two peaks from ^211^At in the two holes. Based on our findings, reducing the thickness of the collimator to increase sensitivity has a minimal effect on the spatial resolution.

**Figure 4:**
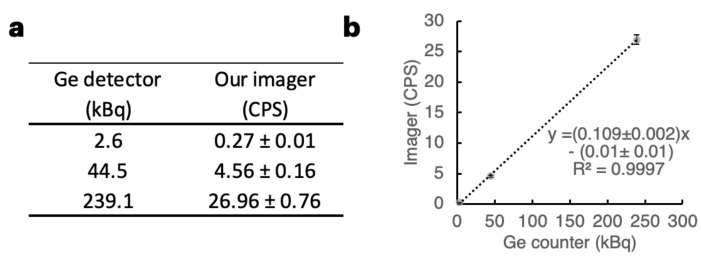
Reference curve for ^211^At. (**a**) Data of the three ^211^At samples regarding the photon count from our imager and dose data using a Ge detector. The count data represent the acquired values ± S.D. (S.D. is derived from ^211^At decay). CPS: count per second. (**b**) Plot illustrating the strong linear correlation between the activity measured using a dose calibrator and the count per second obtained with our imager.

To obtain a reference curve for ^211^At, three sample solutions at three points of ^211^At dosage were prepared. The samples were measured with both our imager (75–85 keV) and a Ge detector (Figure 4a). A strong correlation was observed between the photon counts from our imager and the activity using a Ge detector. This is shown in Figure 4b ( $r^{2}$ = 0.9997).

Table 1 delineates the minimum dose required to achieve the desired accuracy (statistical error) for imaging target tissue within a specified acquisition time. To maintain statistical errors within ±15%, ±10%, and ±5%, at least 44, 100, and 400 photons are necessary, respectively. The minimal count per second (CPS) required was calculated by dividing the required photon counts by the acquisition time. The minimal dose for each acquisition time was then determined by dividing the CPS by the slope of the linear equation in Figure 4b. When an acquisition time is set to 30 min (the acquisition time for this study), an activity over 2.04 kBq is crucial to ensure a statistical error margin of ±5%, which matches the accuracy provided by a dose calibrator. For data requiring a statistical error of 15% or less in 30 min of acquisition time, the estimated minimum accumulation dose is 0.23 kBq.

The effective doses of ^211^At-labeled agents, which are administered without causing serious adverse effects, range from 100 to 1000 kBq. The maximum %IDs to target tumors range from approximately a few percent up to 23% [21,23,24,25,26,27,28]. When referring to these reports, the most challenging scenario for quantitative imaging in terms of administration dose and tumor distribution rate is 100 kBq of administration dose and a tumor distribution rate of 1.0%. Then, if the half-life of ^211^At (7.2 h) is not considered, the accumulation of the drug in the tumor is 1.0 kBq in the scenario. Therefore, the minimum requirement was defined that a 1.0 kBq target can be quantitatively imaged under the conditions allowed in Table 1. The condition with the lowest accuracy and longest acquisition time in Table 1 is that with 15% statistical error and 60 min of acquisition time. The minimum dose required in this condition is 0.11 kBq, and 1.0 kBq is well above this value; thus, quantitative imaging with the required accuracy is possible even in the challenging scenario described above. Therefore, this imaging system can be applied in many cases regarding the %ID of the drug to targets, dosage, and timing of imaging after administration under the conditions of 15% statistical error and 60 min of acquisition time.

### 2.3. Cell Imaging Using ^*211*^At-NaAt Solution

**Figure 5:**
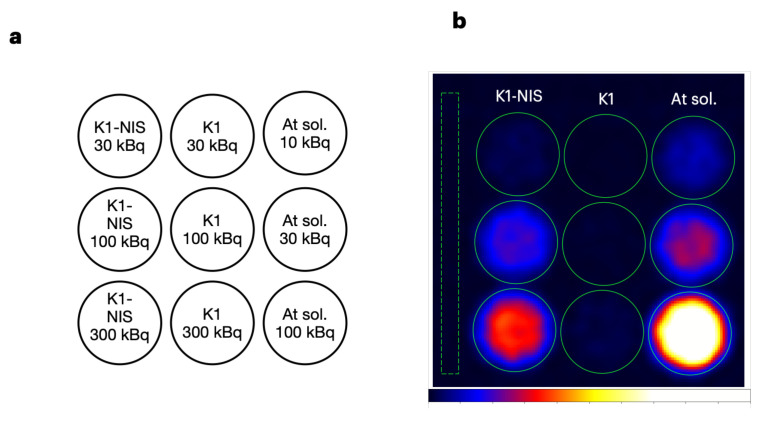
Cellular imaging. (**a**) K1-NIS cells were seeded in the left-column wells, and an equivalent number of K1 cells were seeded in the middle-column wells. No cells were seeded in wells in the right column (the wells for reference). Medium containing the specified activity of ^211^At-NaAt was added, as shown in the figure. After 5 min of incubation, the medium was removed and the cells were washed once with PBS. The wells in the right column contained the medium comprising ^211^At-NaAt. (**b**) Planar image of the well plate. Image was obtained using the ^211^At (^211^Po) X-rays (75–85 keV) with an acquisition time of 30 min. The leftmost dashed rectangle indicates the ROI for the background.

Our previous study revealed that ^211^At-NaAt was effective against sodium iodide symporter (NIS)–expressing thyroid cancer cells [22] and a xenograft model [21]. The NIS, also known as SLC5A5, is responsible for the uptake of $I^{-}$ and ${\mathrm{At}}^{-}$ [29]. K1-NIS cells, which are NIS-transfected K1 cells (human papillary thyroid carcinoma with low expression of NIS), were used in the studies. Kaneda-Nakashima et al. reported that (1) K1 cells display increased ^211^At uptake according to the expression level of NIS (from approximately 0 to 10 kBq per 1.0 × 10^5^ cells), and (2) the therapeutic effect is also observed according to the amount of ^211^At uptake [22]. Watabe et al. reported that the activity concentration of ^211^At in K1-NIS tumors was 22.5 ± 10.4 %ID at 3 h after administration [21]. In the present study, we investigated whether the differences in ^211^At accumulation in K1/K1-NIS cells could be assessed quantitatively from the images obtained with our imager using cultured cells. Accordingly, 3.0 × 10^4^ K1 cells and K1-NIS cells seeded in a 96-well plate were incubated with the medium containing ^211^At-NaAt (30, 100, and 300 kBq to each well; n = 1 each; Figure 5a), washed with PBS, and imaged using our imager with an acquisition time of 30 min. The K1-NIS cells produced images with varying intensities correlating with the dose of ^211^At. Meanwhile, the K1 cells showed a significantly low intensity (Figure 5b). The dose data obtained by setting the region of interest (ROI) on the image of each well demonstrated an ^211^At uptake rate of approximately 11–24% for K1-NIS cells, whereas K1 cells had an uptake rate of less than 1% (Table 2). An image analysis yielded results consistent with the previous studies. The quantitative data obtained from the imaging of wells without cells were in alignment with the dose calibrator data, considering the accuracy of the dose calibrator (±5%) and errors associated with pipetting.

### 2.4. In Vivo Imaging in ^*211*^At Radionuclide Therapy

**Figure 6:**
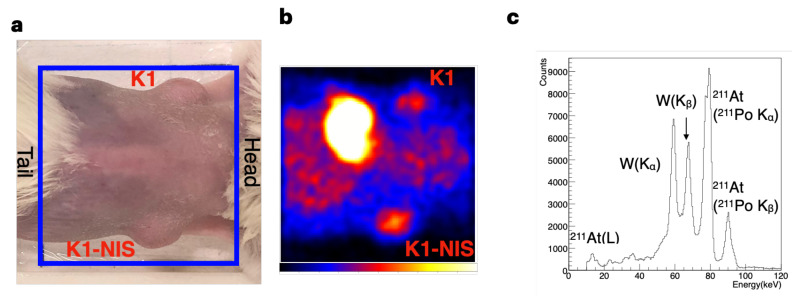
In vivo imaging. (**a**,**b**) Representative mouse image from the back (**a**) and the corresponding planar image of ^211^At (75–85 keV) (**b**). K1 cells and NIS-transfected K1(K1-NIS) cells were transplanted into the left (top) and right (bottom) flanks, respectively. Blue squares indicate the field of observation. Strong accumulation of ^211^At was observed not only in the NIS-expressing tumor but also in the stomach, which expresses NIS. (**c**) Representative spectrum obtained by mouse imaging.

In vivo imaging was performed in the therapeutic experiments using K1 and K1-NIS cells. Cancer-bearing mice with K1 cells transplanted into their left flank and K1-NIS cells transplanted into their right flank (Figure 6a) were administered approximately 1.0 MBq of ^211^At-NaAt through the tail vein. Image data were acquired 1 h after the administration (n = 6). A representative image is shown in Figure 6b, and the images of all mice are shown in Figure S1: all images of the six mice. The spectrum obtained with our imager is shown in Figure 3c. Here, the scattering component from the mouse is not clearly recognizable with reference to Figure 3b, which does not include it.

**Figure 7:**
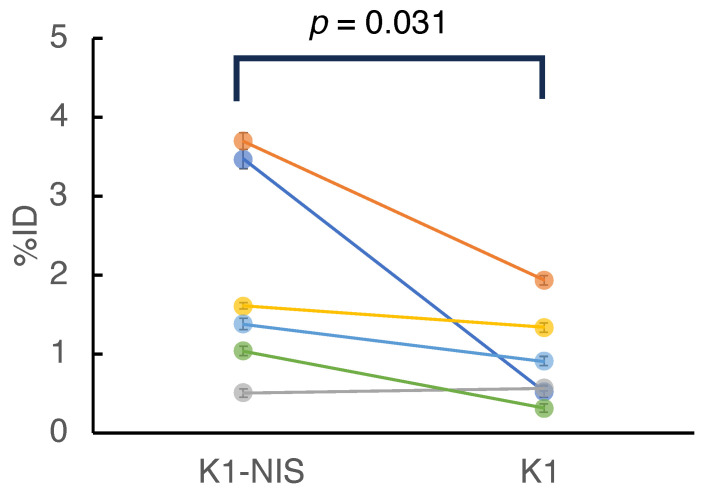
Activity of ^211^At in the tumors (n = 6). Each color indicates each pair of tumors in the mouse. Error bars represent the S.D. arising from the atomic decay of ^211^At. The ^211^At activity (%ID) of K1-NIS tumors was significantly lower than that of K1 tumors (sign test, *p* = 0.031, n = 6).

For further data analysis, a region of interest (ROI) was designated, which corresponded to the tumor site on each image. Detailed data, including doses, counts, and activity for all mice, are available in Table 3. The activity (%ID) of ^211^At in the tumors is shown in Figure 7. In five of the six mice, K1-NIS cell tumors had a higher activity than the K1 cell tumors, with no activity difference found in one case. The activity (%ID) of the two groups (K1 vs. K1-NIS) was significantly different in the sign test (n = 6, K1-NIS > K1, *p* = 0.031).

**Figure 8:**
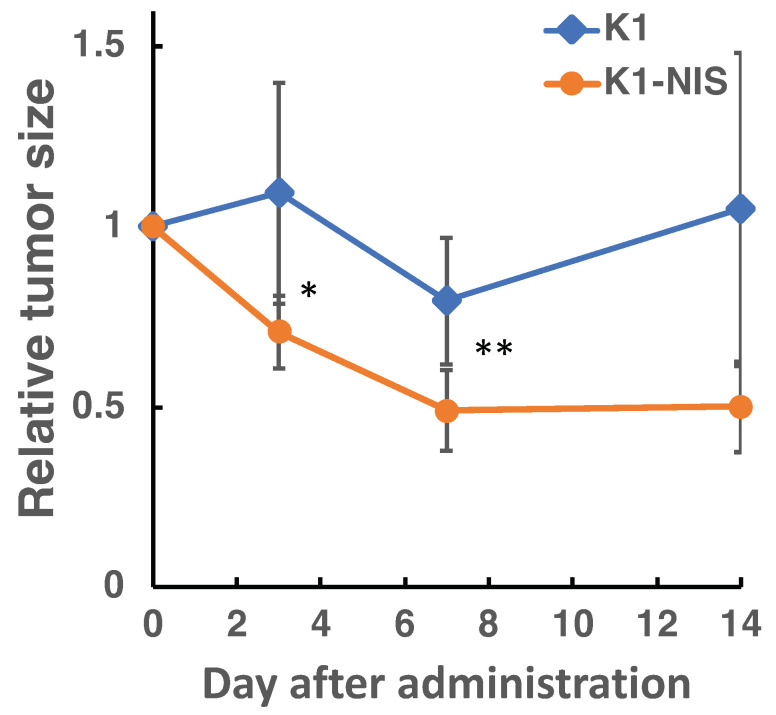
Changes in relative tumor size after treatment. Data are presented as the mean ± S.D., (n = 6, the Student’s *t*-test, *p < 0.05, ** *p* < 0.01).

The time course of tumor size following treatment is shown in Figure 8. The K1-NIS tumors were significantly smaller than those of the K1 tumors at both day 3 and 7 post-treatment (n = 6, the Student’s *t*-test, *p* = 0.015, *p* = 0.005, respectively). This finding aligned with the observed ^211^At activity in the tumors.

## 3. Discussion

By performing in vitro experiments in this study, the performance of our imager in terms of sensitivity, spatial resolution, and energy resolution, was confirmed to meet the requirements for in vivo imaging of ^211^At. Although the experiment was straightforward, we investigated the accumulation of ^211^At in cells using imaging. An image that reflects the activity of ^211^At and that of accumulated ^211^At according to the expression level of NIS (the target molecule of the therapeutic agent in this study) could be obtained. The capability to obtain both imaging data on drug accumulation and quantitative data on the drug in cell-based experiments is of high utility in drug discovery. The accumulation of a tracer in cells can be measured by a γ -counter using cell suspensions. However, this method is somewhat complicated because it requires the washing and centrifugation of samples and the removal of buffer multiple times, depending on the number of specimens. In quantitative cell imaging, a series of operations can be easily and quickly performed because the cells are attached to the culture plate, and both the activity and the image can be obtained. Furthermore, if the cells are fixed in formalin, quantitative imaging can be followed by fluorescent immunostaining analysis. For instance, Kaneda-Nakashima et al. reported a positive correlation between an accumulated dose of ^211^At and the number of γ H2AX foci in immunofluorescence images [22], which is a marker molecule of DNA double-strand break. In this study, the cell accumulation dose per applied dose and the number of γ H2AX foci per applied dose were obtained by individual experiments. Our imaging system allows these experiments to be performed as a sequence of experiments using the same cells. Simplicity and low cost are important in drug screening. This imaging system satisfies these criteria.

A summary of the requirements for the system and the achieved results is shown in Table 4. In the animal experiment, the activity of ^211^At in NIS-positive and NIS-negative xenografts was evaluated, and the correlation with the treatment response could be evaluated. The difference in ^211^At activity in the K1-NIS and K1 tumors was less than anticipated, considering the findings of the previous study, where the ^211^At activity concentration in K1-NIS tumor was 22.5 ± 10.4 %ID of ^211^At, despite being measured 3 h after injection [21]. However, even under such circumstances, a correlation could be found between the ^211^At activity in the tumors and treatment outcome. Administering the same dose does not always guarantee identical drug distribution within tumors. Hence, as demonstrated in this study, the activity of therapeutic agents within tumors should be evaluated using quantitative imaging, ensuring a more effective assessment of drug efficacy.

There is growing interest in the use of ^211^At as an α -emitter for targeted radionuclide therapy [15]. For instance, ^211^At-NaAt [21], ^211^At-labeled antihuman epidermal growth factor receptor 2 antibody [23], ^211^At-astato-benzylguanidine [24], ^211^At-phenylalanine [25], ^211^At-prostate-specific membrane antigen [26], ^211^At-octreotide [27], and ^211^At- α -methyl-l-tyrosine [28] have been reported as promising agents. In these studies, the minimum effective doses of ^211^At-labeled agents are 100 kBq, and the minimum %ID to target tumors is approximately a few percent. If high accuracy is not required, many cases can be accommodated by our system. On the other hand, for agents that accumulate in tumors to a lesser extent, imaging conducted 24 h after administration, or situations requiring high-accuracy imaging of low-activity targets in a short acquisition time, may not be suitable. To obtain the absorbed dose, it is necessary to obtain the dose in tumors after 24 h of administration. However, 24 h is more than three half-lives of that of ^211^At (7.2 h), reducing it to less than one-eighth of the original activity. Therefore, to obtain absorbed doses with high accuracy, the sensitivity of the device needed to be improved.

Sensitivity is not the only important aspect of quantitative imaging. This study underscores the necessity of accurately quantifying radiopharmaceuticals for drug development. A method to achieve this is to use sensors with a high energy resolution. The energy resolution of a CdTe sensor is higher than those of CdZnTe sensors and the NaI(Tl) scintillator (1.6%, 5.3%, and 8.7% at 140 keV, respectively) [14], which are currently used in SPECT imaging. This study demonstrates that the high energy resolution of the CdTe sensor enables differentiation between X-rays from ^211^At and fluorescent X-rays from tungsten, thereby achieving superior quantitative accuracy. Another approach is to mitigate noise signals, such as scattering components, which increase with an increase in body sizes. Therefore, noise reduction methods are crucial. We have developed a method to remove noise signals by applying a spectroscopic analysis method to spectral data obtained from CdTe sensors [30].

Our planar imager is bench-top-sized and is markedly smaller than small animal SPECT. The cost for its development and maintenance is less than that of SPECT. Moreover, the operational simplicity of this imager—requiring users to merely place the mouse on the imager for image acquisition—ensures minimal interference during treatment observation experiments. However, unlike SPECT, our imager lacks 3D information. Depending on the position of the target tissue, overlaps with other tissues can occur in the image, potentially complicating accurate activity acquisition. In scenarios where the target tissue activity is overshadowed by surrounding tissue activity, precise estimates become challenging. Nonetheless, given specific requirements, our planar imaging system remains a viable choice. The key areas for enhancement in the planar imaging system include enlarging the field of view (FOV) and enhancing the sensitivity. Currently, the imager’s FOV accommodates only half of a mouse’s body. This necessitates repositioning to capture the full-body image. An expanded FOV will enable whole-body imaging in a single session. Moreover, heightened sensitivity could shorten the imaging time, thereby improving the efficiency of cellular imaging workflows.

## 4. Conclusions

We developed a system to analyze the intratumoral drug distribution of the therapeutic candidate ^211^At-NaAt in radiotheranostics via in vivo imaging and confirmed its utility. This imager enabled us to assess drug distribution and treatment outcome in the same mice. Moreover, our data indicate the utility of quantitative imaging in drug development via cell imaging using ^211^At-labeled agents. However, it is necessary to accumulate data on the utility of quantitative imaging by performing quantitative imaging of many therapeutic agents. The limitations of this system are that the sensitivity is not sufficient for imaging low-activity samples with high accuracy and that the FOV does not cover the entire mouse body. Theranostics is a coined term for “therapy” and “diagnostics”. We hope that our imaging system will contribute to the advancement of this field from a “diagnostic” perspective.

## 5. Materials and Methods

### 5.1. Imager Setup and Its Performance

The basic imaging system configuration was reported previously [16]. The imager consists of a CdTe semiconductor detector imaging sensor, a parallel-hole collimator made of tungsten with a 3D metal printer (Toray Precision Co., Ltd., Shiga, Japan), a Peltier cooling system, a readout system, and a housing. The density of the collimator is measured to be 19.13 g cm^−3^, which is consistent with that of tungsten metal. The CdTe sensor is a double-sided strip detector (CdTe-DSD), where imaging is based on coincidence detection on signals from strips on both sides of the detector. The CdTe-DSD is a square-shape detector with a thickness of 32 mm on each side. The thickness of the CdTe crystal is 0.75 mm. The detection efficiency is 85% for 59.5 keV. The detector has an energy resolution of 1–2 keV for 10–100 keV and a position resolution of 250 μm. In this study, the collimator thickness was changed from 20 mm in the previous study to 10 mm. Collimator efficiency (g) was calculated by substituting the septa thickness (*t*), collimator thickness (*l*), and hole diameter (*d*) into the following formula [16,31]:

(1) $$g=0.26^{2}{\left(\frac{d}{l}\right)}^{2}\frac{d^{2}}{{\left(d+t\right)}^{2}}$$

The energy window of ^211^At was set to 75–85 keV. Spatial resolution was evaluated using a Derenzo phantom with holes of diameters of 1.2, 1.6, 2.4, 3.2, 40, and 4.8 mm. Each hole was filled with an ^211^At solution. Three sample solutions were prepared at three points of dosage to obtain a reference curve for ^211^At. The samples were measured with both our imager and a Ge detector (BE2020, Mirion Technologies (Canberra), Zellik Belgium), CT, USA). The reference curve was obtained from photon count data and dose data using linear regression.

### 5.2. Preparation of ^*211*^At Solutions

The ^211^At solutions were prepared as described previously [21]. Briefly, ^211^At was procured from the RIKEN Nishina Center for Accelerator-Based Science. ^211^At was produced by the ^209^Bi( α , 2n)^211^At reaction, and then separated and purified using a dry distillation method. ^211^At was dissolved in distilled water containing 1.2% (*w*/*v*) ascorbic acid (IWAKI & CO., Ltd., Tokyo, Japan) as a reducing agent.

### 5.3. Cell Culture

K1 cells (human papillary thyroid carcinoma) were provided by the European Collection of Authenticated Cell Cultures. K1-NIS cells were obtained via transfection using the human SLC5A5 (NIS) gene clone (OriGene, Rockville, MD), as described previously [21]. K1 and K1-NIS cells were maintained in culture medium, D-MEM (Nacalai Tesque, Inc. Kyoto, Japan):Ham F12 (Nacalai Tesque):MCDB 105 (Cell Applications, Inc., San Diego, CA, USA) (2:1:1) supplemented with 2 mM glutamine (Nacalai Tesque) and 10% heat-inactivated fetal bovine serum (Corning, NY, USA).

### 5.4. Preparation of Animals

NOD-SCID mice (male, 4 weeks old) were purchased from Charles River Japan, Inc. (Atsugi, Japan). Animals were housed under a 12 h light/12 h dark cycle and granted free access to food and water. Tumor xenograft models were established in NOD-SCID mice by subcutaneously injecting K1-NIS cells into the upper left-flank and K1 cells into the upper-right flank. Each injection comprised 1.0 × 10^7^ cells suspended in 0.2 mL of a 1:1 mixture of culture medium and Matrigel (BD Biosciences, San Jose, CA, USA). All animal experiments complied with the guidelines of the Institute of Experimental Animal Sciences. The protocol was approved by the Animal Care and Use Committee of the Osaka University Graduate School of Medicine (Approval No. 30-103-008, approval date 20 February 2019). Tumor size was monitored, and mice were euthanized after 2 weeks via deep anesthesia with isoflurane inhalation.

### 5.5. Imaging Conditions for Cellular and Animal Imaging

For cellular imaging, 3.0 × 10^4^ of K1 cells and K1-NIS cells were seeded in each well of 96-well plates 1 day prior to the experiment. The cells were incubated with medium containing 30, 100, and 300 kBq of ^211^At-NaAt, which was measured with a dose calibrator (Curie meter IGC-8B, Hitachi, Tokyo, Japan) with an accuracy of ±5%, for 5 min; rinsed once with PBS; and imaged with our imager (acquisition time of 30 min). The accumulated ^211^At activity in the cells were determined by subtracting the background activity from the activity in the well ROI. For animal imaging, mice were anesthetized with isoflurane (Viatris Pharmaceuticals Japan, Tokyo, Japan; induction 3%, maintenance 1.5%) during the administration of the ^211^At solutions and imaging. Each cancer-bearing mouse was administered approximately 1.0 MBq of ^211^At solution (measured with a dose calibrator) through the tail vein. One hour postadministration, mice were imaged for 30 min. The accumulated ^211^At activity in the tumors was determined by subtracting the background activity from the activity in the tumor ROI. The background per area was estimated according to the activity of the area surrounding the tumors. In the comparison of ^211^At activity in K1-NIS/K1 tumors using the sign test, if the activity difference in each mouse exceeded the statistical error due to At decay, + or − was assigned, and those within the statistical error were treated as having no difference. Statistic analysis was carried out using the latest version of ROOT 6 (Cern, Meyrin, Switzerland).

## Figures and Tables

**Table 1:** Required minimal dose accumulation (kBq) to a target tissue according to accuracy and acquisition time.

Accuracy (Statistical Error)	Acquisition Time (min)			
	10	20	30	60
5%	6.12	3.06	2.04	1.02
10%	1.53	0.76	0.51	0.25
15%	0.68	0.34	0.23	0.11

**Table 2:** Cellular imaging data. Photon counts in cells were obtained from the ROIs assigned to each well. Cell activity was determined by converting the count data. CPS: count per second; n.d.: not detected; n.a.: not available.

Well	Loaded Activity (kBq)	CPS	Activity (kBq)	Uptake Rate (%)
K1-NIS cell	300	4.77	44.07	14.69
	100	2.58	23.94	23.94
	30	0.35	3.34	11.13
K1 cell	300	0.28	2.7	0.9
	100	0.08	0.83	0.83
	30	n.d.	n.a.	n.a.
Reference (no cell)	100	11.58	106.84	-
	30	3.3	30.55	-
	10	1.11	10.36	-

**Table 3:** Activity of ^211^At in tumors. Data represent the acquired values ± S.D. (S.D. is derived from ^211^At decay).

Mouse No.	Dose (MBq)	K1-NIS		K1	
		Activity (kBq)	%ID (%)	Activity (kBq)	%ID (%)
1	1.16	40.34 ± 1.31	3.47 ± 0.11	6.13 ± 0.69	0.53 ± 0.06
2	1.07	39.68 ± 1.29	3.7 ± 0.12	20.81 ± 0.9	1.94 ± 0.08
3	1.07	5.49 ± 0.44	0.51 ± 0.04	6.14 ± 0.59	0.57 ± 0.06
4	1.05	16.91 ± 0.7	1.61 ± 0.07	14.07 ± 0.65	1.34 ± 0.06
5	1.04	14.32 ± 0.62	1.38 ± 0.06	9.48 ± 0.52	0.91 ± 0.05
6	1.07	11.08 ± 0.56	1.04 ± 0.05	3.46 ± 0.36	0.32 ± 0.03

**Table 4:** Summary of requirements and achieved results.

	Requirements	Achieved Results
**Sensitivity**	Is quantitative imaging of many therapeutics possible concerning dose and tumor accumulation rate?	The system is capable of handling many cases as detailed in Table 1 and the results.
**Spatial resolution**	Is spatial resolution sufficient to distinguish mouse organs?	The spatial resolution is better than 1.6 mm, which is sufficient to identify small organs such as the thyroid gland.
**Energy resolution**	Is the energy resolution sufficient to distinguish it from noise signals?	Its high energy resolution makes it possible to distinguish between X-rays from ^211^At and tungsten fluorescent X-rays.

## Data Availability

All data generated or analyzed in this study are included in this published article and the Supplementary Information file.

## Supplementary Materials

The following supporting information can be downloaded at www.mdpi.com/xxx/s1, Figure S1: All images of the six mice.

## Abbreviations

The following abbreviations are used in this manuscript:

SPECT	single-photon emission computed tomography
NIS	sodium iodide symporter
SN-ratio	signal-to-noise ratio
CdTe	cadmium telluride
CPS	count per second
ROI	region of interest
%ID	percentage of injected dose
FOV	field of view
