# Supplementary material for: Development and Utility of an Imaging System for Internal Dosimetry of Astatine-211 in Mice
Source: Bioengineering (Basel). 2023 Dec 26;11(1):0. doi: 10.3390/bioengineering11010025 (PMC11154565; doi:10.3390/bioengineering11010025)
Supplement: Supplementary file 1 [file bioengineering-11-00025-s001.zip › bioengineering-2776932-supplementary.pdf]

Supplementary Information

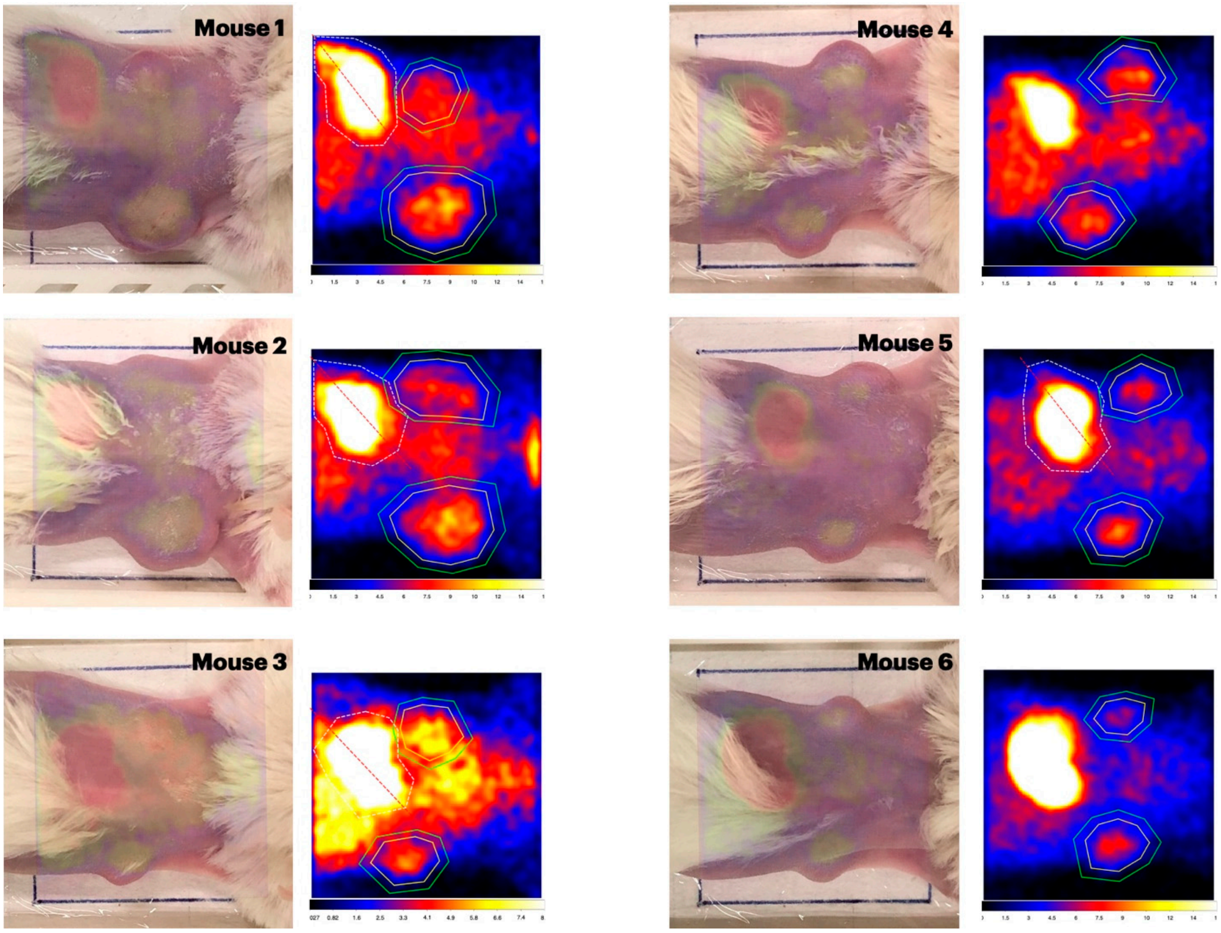

Figure S1. All images of the six mice. The ROI was placed on the image as an inner circle around the tumor. The outer circle was established to obtain the activity of the non-tumorous tissue around the tumor for background subtraction.
